# Whole-genome sequences of four *Vibrio* species isolated from shrimp in local markets of Bangladesh

**DOI:** 10.1128/mra.01096-25

**Published:** 2025-11-10

**Authors:** Mohammad Sharif Uddin, Kazi Chamonara, Sumaia Sultana, Sajedul Islam, Afifa Siddiqua, Md. Habib Ullah Masum

**Affiliations:** 1 Department of Microbiology, Noakhali Science and Technology University (NSTU) 378872, Noakhali, Bangladesh; 2 Department of Environmental Biotechnology, Faculty of Biotechnology and Genetic Engineering, Chattogram Veterinary and Animal Sciences University (CVASU) 130057, Chattogram, Bangladesh; 3 Department of Physiology, Biochemistry and Pharmacology, Chattogram Veterinary and Animal Sciences University (CVASU) 130057, Chattogram, Bangladesh; 4 Department of Genomics and Bioinformatics, Faculty of Biotechnology and Genetic Engineering, Chattogram Veterinary and Animal Sciences University (CVASU) 130057, Chattogram, Bangladesh; Montana State University, Bozeman, Montana, USA

**Keywords:** zoonotic infections, fish microbiology

## Abstract

Bangladesh’s aquaculture is currently faced with *Vibrio* contamination, which poses a threat to public health, exports, and product quality. This research provides a detailed molecular characterization of *Vibrio* sp. found in shrimp through the use of Oxford Nanopore sequencing, ensuring precise identification and enhancing shrimp quality and food security.

## ANNOUNCEMENT

The aquaculture sector in Bangladesh substantially contributes to the national economy and food security, which accounts for 3.52% of GDP and 26.37% of agricultural GDP, ranking Bangladesh third in inland capture and fifth in aquaculture globally (1). Among the fish species, shrimp are significant contributors to export revenue. Nevertheless, the consumption of contaminated shrimp poses a significant risk of foodborne infections, thereby threatening both public health and the sustainability of this sector (2). The contamination frequently occurs due to inadequate practices in processing, preservation, and storage, which provide favorable growth conditions for food spoilage bacteria, like *Vibrio* spp. (3–5). Twelve species of *Vibrio* are pathogenic to humans, with *V. parahaemolyticus* and *V. cholerae* being the most prevalent (6). These pathogens also pose significant zoonotic and public health risks (4). Additionally, the shrimp export sector in Bangladesh encounters significant challenges due to quality decline stemming from *Vibrio* contamination (7). However, prompt identification of pathogenic *Vibrio* species can enhance shrimp quality, export prospects, and customer safety (7, 8). Using Oxford Nanopore sequencing, this study reveals the molecular profiles of *V. cholerae* and *V. parahaemolyticus* from shrimp samples of Noakhali district, filling gaps in *Vibrio* molecular characterization.

The shrimp samples were enriched in alkaline peptone water (at 37°C), then incubated onto thiosulfate–citrate–bile salts–sucrose (TCBS) agar plates (for 18–24 h at 37°C) (9). Presumptive *Vibrio* isolates were initially identified based on morphological traits, followed by confirmation through polymerase chain reaction (PCR) aimed at the genus-specific groEL gene. Subsequent culture of the isolates in nutrient agar was applied for the total DNA extraction by using QIAamp DNA Mini Kit following the manufacturer’s instructions and quantified using a Qubit four fluorometer. In advance of DNA sequencing, a native barcoding kit (SQK.NBD.114.24) was employed for library preparation (full length 16S rRNA, V1-V9), and the sequencing run was executed on the Oxford Nanopore MinION Mk1C platform utilizing the FLO-MIN114 (R10.4.1) flow cell. The MINKNOW software (v.1.11.5) was utilized for gathering information. The Guppy (v.6.3.2) was employed for basecalling the MinION Mk1C sequencing data (FAST5 files) to provide pass reads (FASTQ format) with a mean quality score above 9. The adapter and barcode sequences were trimmed using Porechop (v.0.2.4) (10), and low-quality bases (q < 10) were removed with Nanofilt (11). The quality-trimmed reads were assembled into contigs using Flye (v.2.9.1-b1780) through the *de novo* assembly workflow (12).

The Type (Strain) Genome Server (TYGS) was utilized to identify the species (13). The potential contamination within the genome and its completeness was assessed using ConEst16S (14), ChecKM (v.1.2.4) (15), BUSCO (v.6.0.0) (16), and QUAST (v.5.2.0) (17). The average nucleotide identity (ANI) and digital DNA:DNA hybridization (dDDH) were determined using the OrthoANI (v. 0.93.1) tool available on the Ezbiocloud server (18) and the Genome-to-Genome Distance Calculator tool (v.3.0), respectively (19). The genome was annotated using three distinct tools: Prokka (1.14.6) (20), Rapid Annotations using Subsystems Technology (RAST) Tool Kit (v.1.3) (21), and NCBI Prokaryotic Genome Annotation Pipeline (PGAP) (v.6.10) (22, 23) (Table 1).

**TABLE 1:** Genome features of four *Vibrio* isolates from shrimp in local markets of Noakhali, Bangladesh

Feature	Strain			
	SU37A	SU91A	SU109	SU129B
Species	*Vibrio parahaemolyticus*	*Vibrio parahaemolyticus*	*Vibrio cholerae*	*Vibrio cholerae*
GenBank accession no.	JBQRUG000000000.1	JBQWLY000000000.1	JBQWLZ000000000.1	JBQWMA000000000.1
SRA accession no.	SRR34709126	SRR34709125	SRR34709124	SRR34709123
Genome size (Mbp)	5037599	5156510	4042637	4053857
No. of reads	820864	1195744	385028	819028
Genome coverage (x)	161.5	209.7	109.1	247.4
Coarse consistency (%)	99.9	99.7	99.8	99.7
Fine consistency (**%**)	98.7	91.9	98.5	96.7
Completeness (**%**)	97.04	86.55	97.63	93.08
No. of contigs	3	3	2	3
N50 (bp)	3252908	3376922	2870474	2888403
No. of L50	1	1	1	1
GC content (**%**)	45.43	45.34	47.67	47.63
ANI score (**%**)	98.49	98.44	98.35	98.34
dDDH (**%**)	86.60	86.10	85.10	85.60
No. of Genes (Prokka, RAST, PGAP)	4696, 4850, 4632	5155, 5383, 4740	3695, 3878, 3743	3742, 3938, 3707
CDS (Prokka, RAST, PGAP)	4523, 4681, 4459	4989, 5216, 4571	3558, 3743, 3602	3606, 3804, 3568
tRNA (Prokka, RAST, PGAP)	135, 132, 132	128, 130, 128	105, 106, 106	104, 104, 104
rRNA (Prokka, RAST, PGAP)	37, 37, 37	37, 37, 37	31, 29, 31	31, 30, 31
Proteins with functional assignments	3746	4157	3030	3106
Antibiotic resistance gene (CARD, PATRIC)	8, 36	11, 44	6, 33	6, 32

## Data Availability

All sequencing data generated in this study have been deposited in the NCBI BioProject database under accession number PRJNA1294324, and are publicly available through the Sequence Read Archive (SRA) (SRR34709126, SRR34709125, SRR34709124, and SRR34709123). The complete genome data have been submitted to NCBI GenBank with the accession numbers JBQRUG000000000.1, JBQWLY000000000.1, JBQWLZ000000000.1, and JBQWMA000000000.1. The RAST annotation files have been deposited in the figshare as File S1 (https://doi.org/10.6084/m9.figshare.30390940.v1).

## References

[1] DoF. 2020. Yearbook of fisheries statistics of Bangladesh, p 141. Department of fisheries. Bangladesh: ministry of fisheries and livestock.

[2] Yaashikaa PR, Saravanan A, Kumar PS. 2016. Isolation and identification of Vibrio cholerae and Vibrio parahaemolyticus from prawn (Penaeus monodon) seafood: preservation strategies. Microb Pathog 99:5–13. doi:10.1016/j.micpath.2016.07.014 27457973

[3] Vu TTT, Alter T, Huehn S. 2018. Prevalence of Vibrio spp. in retail seafood in Berlin, Germany. J Food Prot 81:593–597. doi:10.4315/0362-028X.JFP-17-366 29517352

[4] Austin B. 2010. Vibrios as causal agents of zoonoses. Vet Microbiol 140:310–317. doi:10.1016/j.vetmic.2009.03.015 19342185

[5] Oramadike C, Ogunbanwo ST. 2015. Prevalence and antimicrobial susceptibility of Vibrio parahaemolyticus isolated from seafoods in Lagos lagoon Nigeria. Cogent Food Agric 1:1041349. doi:10.1080/23311932.2015.1041349

[6] Bonnin-Jusserand M, Copin S, Le Bris C, Brauge T, Gay M, Brisabois A, Grard T, Midelet-Bourdin G. 2019. Vibrio species involved in seafood-borne outbreaks (Vibrio cholerae, V. parahaemolyticus and V. vulnificus): review of microbiological versus recent molecular detection methods in seafood products. Crit Rev Food Sci Nutr 59:597–610. doi:10.1080/10408398.2017.1384715 28956623

[7] Nilla SS, Mustafa MG, Ahsan DA, Rahman Khan MM, Rahman Khan MA. 2012. Bacterial abundance in indian white shrimp, Penaeus indicus collected from two different market conditions of Dhaka city. Dhaka Univ J Biol Sci 21:29–38. doi:10.3329/dujbs.v21i1.9742

[8] Siddique AB, Moniruzzaman M, Ali S, Dewan MN, Islam MR, Islam MS, Amin MB, Mondal D, Parvez AK, Mahmud ZH. 2021. Characterization of pathogenic Vibrio parahaemolyticus isolated from fish aquaculture of the Southwest coastal area of Bangladesh. Front Microbiol 12:635539. doi:10.3389/fmicb.2021.635539 33763050 PMC7982743

[9] WHO. 2016. Selection and application of methods for the detection and enumeration of human-pathogenic halophilic Vibrio spp. in seafood: guidance, on World Health Organization & Food and Agriculture Organization of the United Nations. https://www.who.int/publications/i/item/9789241565288.

[10] Wick R, JJAogcrP V. 2018. Porechop: adapter trimmer for oxford nanopore reads. Available from: https://manpages.ubuntu.com/manpages/jammy/man1/porechop.1.html

[11] De Coster W, D’Hert S, Schultz DT, Cruts M, Van Broeckhoven C. 2018. NanoPack: visualizing and processing long-read sequencing data. Bioinformatics 34:2666–2669. doi:10.1093/bioinformatics/bty149 29547981 PMC6061794

[12] Kolmogorov M, Yuan J, Lin Y, Pevzner PA. 2019. Assembly of long, error-prone reads using repeat graphs. Nat Biotechnol 37:540–546. doi:10.1038/s41587-019-0072-8 30936562

[13] Meier-Kolthoff JP, Göker M. 2019. TYGS is an automated high-throughput platform for state-of-the-art genome-based taxonomy. Nat Commun 10:2182. doi:10.1038/s41467-019-10210-3 31097708 PMC6522516

[14] Lee I, Chalita M, Ha SM, Na SI, Yoon SH, Chun J. 2017. ContEst16S: an algorithm that identifies contaminated prokaryotic genomes using 16S RNA gene sequences. Int J Syst Evol Microbiol 67:2053–2057. doi:10.1099/ijsem.0.001872 28639931

[15] Parks DH, Imelfort M, Skennerton CT, Hugenholtz P, Tyson GW. 2015. CheckM: assessing the quality of microbial genomes recovered from isolates, single cells, and metagenomes. Genome Res 25:1043–1055. doi:10.1101/gr.186072.114 25977477 PMC4484387

[16] Manni M, Berkeley MR, Seppey M, Zdobnov EM. 2021. BUSCO: assessing genomic data quality and beyond. Curr Protoc 1:e323. doi:10.1002/cpz1.323 34936221

[17] Gurevich A, Saveliev V, Vyahhi N, Tesler G. 2013. QUAST: quality assessment tool for genome assemblies. Bioinformatics 29:1072–1075. doi:10.1093/bioinformatics/btt086 23422339 PMC3624806

[18] Lee I, Ouk Kim Y, Park SC, Chun J. 2016. OrthoANI: An improved algorithm and software for calculating average nucleotide identity. Int J Syst Evol Microbiol 66:1100–1103. doi:10.1099/ijsem.0.000760 26585518

[19] Meier-Kolthoff JP, Auch AF, Klenk H-P, Göker M. 2013. Genome sequence-based species delimitation with confidence intervals and improved distance functions. BMC Bioinformatics 14:60. doi:10.1186/1471-2105-14-60 23432962 PMC3665452

[20] Seemann T. 2014. Prokka: rapid prokaryotic genome annotation. Bioinformatics 30:2068–2069. doi:10.1093/bioinformatics/btu153 24642063

[21] Aziz RK, Bartels D, Best AA, DeJongh M, Disz T, Edwards RA, Formsma K, Gerdes S, Glass EM, Kubal M, et al.. 2008. The RAST Server: rapid annotations using subsystems technology. BMC Genomics 9:75. doi:10.1186/1471-2164-9-75 18261238 PMC2265698

[22] Tatusova T, DiCuccio M, Badretdin A, Chetvernin V, Nawrocki EP, Zaslavsky L, Lomsadze A, Pruitt KD, Borodovsky M, Ostell J. 2016. NCBI prokaryotic genome annotation pipeline. Nucleic Acids Res 44:6614–6624. doi:10.1093/nar/gkw569 27342282 PMC5001611

[23] Li W, O’Neill KR, Haft DH, DiCuccio M, Chetvernin V, Badretdin A, Coulouris G, Chitsaz F, Derbyshire MK, Durkin AS, Gonzales NR, Gwadz M, Lanczycki CJ, et al.. 2021. RefSeq: expanding the prokaryotic genome annotation pipeline reach with protein family model curation. Nucleic Acids Res 49:D1020–D1028. doi:10.1093/nar/gkaa1105 33270901 PMC7779008

